# Evaluation of MRI Denoising Methods Using Unsupervised Learning

**DOI:** 10.3389/frai.2021.642731

**Published:** 2021-06-04

**Authors:** Marc Moreno López, Joshua M. Frederick, Jonathan Ventura

**Affiliations:** ^1^ Department of Computer Science, University of Colorado Colorado Springs, Colorado Springs, CO, United States; ^2^ Department of Computer Science and Software Engineering, California Polytechnic State University, San Luis Obispo, CA, United States

**Keywords:** deep learning, denoising, k-Space, MRI, unsupervised

## Abstract

In this paper we evaluate two unsupervised approaches to denoise Magnetic Resonance Images (MRI) in the complex image space using the raw information that k-space holds. The first method is based on Stein’s Unbiased Risk Estimator, while the second approach is based on a blindspot network, which limits the network’s receptive field. Both methods are tested on two different datasets, one containing real knee MRI and the other consists of synthetic brain MRI. These datasets contain information about the complex image space which will be used for denoising purposes. Both networks are compared against a state-of-the-art algorithm, Non-Local Means (NLM) using quantitative and qualitative measures. For most given metrics and qualitative measures, both networks outperformed NLM, and they prove to be reliable denoising methods.

## 1 Introduction

Magnetic Resonance Imaging, MRI, is one of the most widely used imaging techniques, as it provides detailed information about organs and tissues in a completely non-invasive way. In MRI, data needed to generate images is directly sampled from the spatial frequency domain; however, the quality of this data can be deteriorated by several thermal noise sources and artifacts. Noise in MRI is of major consequence as it can mislead and result in inaccurate diagnoses of patients. In addition to visually corrupting the recovered images, noise is also an obstacle when conducting quantitative imaging on the MRI. The utility of MRI decreases if a region or specific tissue suffers from a low signal to noise ratio. Thus, there is a necessity for an efficient MRI reconstruction process, where denoising methods are applied to noisy images in order to improve both qualitative and quantitative measures of MRI.

Additionally, in the case of *in vivo* MRI, noise is implicit to the acquisition process. When taking an MRI of a living subject, there are multiple noise factors. All other factors withheld, the MR machine has an innate noise component when acquiring an image due to a thermal factor. Another source of thermal noise is inversely proportional to the amount of time that the subject stays inside the MR machine, and while in the machine the subjects movements also contribute to the thermal noise. Finally, the patient’s body temperature and the thermal factor from the MR machine is another key element, specially since a long exposure inside the MR machine could lead to an increase in body temperature, web (2017).

Thus, when training a MRI denoiser, no ground truth is available for the training procedure. Likewise, due to previously discussed movement of the subject, two independent samples for denoising strategies as used by Lehtinen et al. (2018) cannot be reasonably obtained. Thus either synthetic data needs be generated for supervised learning or unsupervised and self-supervised strategies must be employed. As such, we evaluate self-supervised solutions to MRI denoising. Deep self-supervised image denoisers have been seeing recent success for general image denoising tasks, and provide robust denoisers without requiring access to denoised images. Self-supervised denoisers generally under-perform supervised techniques, but arise naturally in cases like MRI, where pure supervised learning is infeasible.

While deep learning has seen success in many areas, there is a lack of methods focused on denoising MRI. Additionally, many traditional techniques denoise MRI in the magnitude space, dismissing the innate spatial frequency information the MRI contain. Most of the MRI denoising methods available use a supervised approach where they use the original MRI as ground truth. We wanted to explore an unsupervised approach using the complex image space, where no ground truth data is needed. Therefore, we will compare two unsupervised denoising approaches that denoise MRI in the spatial frequency space, competing with the more classical and widely used denoising methods.

## 2 Materials and Methods

### 2.1 Related Work

Previous attempts on MRI denoising can be categorized in three different ways: traditional methods, supervised learning, and unsupervised learning.

#### 2.1.1 Traditional Methods

Traditional MRI denoising techniques are generally based on filtering, transformations, or statistical methods such as Mohan et al. (2014). Three of the most widely-used methods currently are bilateral filtering by Tomasi and Manduchi (1998), non-local means by Buades et al. (2005), and BM3D by Dabov et al. (2007).

The bilateral filter presented by Tomasi and Manduchi (1998) is an edge preserving non-iterative method. When applied to an image, it uses a low-pass denoising kernel which adjusts to the original image spatial distribution of pixel-values. This helps preserve the edges while denoising the image. In the presence of sharp transitions, the kernel is weighted according to this transition. This behavior is modeled by a convolution of the intensity values of the image and a non-linear weighting function.

Non-local means, Buades et al. (2005), or NLM, uses the self spatial similarities that natural images have. It exploits the redundancy of the neighborhood pixels to remove the noise. The simplicity of this filter consists of using those similarities to find similar patches on the rest of the image to the patch being denoised. This is known as neighborhood filtering. NLM assigns confidence weights based on similarity to the original patch and its distance from the center of the observed patch. The main issue with NLM is that since it relies on a large space search, it can create a bottleneck in terms of computation.

BM3D, Dabov et al. (2007), is a robust algorithm that has several parameters that can be modified in order to achieve the best denoising. It is an extension of NLM, in the sense that it uses spatial similarities within the image. It starts by searching for patches with similar intensities to the patch that is being denoised. A 3D matrix containing the size of the patch and the aggregated patches is built. Then, a 3D transform is applied. So as to remove high frequency noises, the transform space is filtered and thresholded. Finally, a denoised 3D block is yielded by doing the inverse transformation. To recover the original array, weights are assigned to every patch. These weights are based on the variance and distance of the patch.

#### 2.1.2 Supervised Learning

One of the most well-known approaches for supervised denoising, DnCNN, is presented by Zhang et al. (2017). Their method uses feed-forward Convolutional Neural Networks, CNN. In order to improve both algorithm speed and performance, they use residual modules and batch normalization. This makes their network unique. Also, it does not need to know the level of noise. So, it can perform blind Gaussian denoising.

Bermudez et al. (2018) implemented an autoencoder with skip connections. To test their method, they added Gaussian noise to a T1-weighted brain MRI dataset from healthy subjects. Benou et al. (2017) worked on spatio-temporal denoising of brain MRI using ensembles of deep neural networks. Each network is trained on a different variations of SNR. By doing this, they generate different hypothesis and then select the most likely one to generate a clean output curve using a classification network. This method presented better denoising results than those presented by Gal et al. (2010), where they use a dynamic NLM method, and they were also better than the results presented by Vincent et al. (2010), where they use stacked denoising autoencoders. An interesting approach is presented by Jiang et al. (2018). They use a multi-channel DnCNN to denoise Rician noise in magnitude MRI instead of Gaussian noise. They test their network for both known and unknown levels of noise, which allows them to create a more general model. Finally, Tripathi and Bag (2020) present a CNN with residual learning to denoise synthetic brain MRI. They use five different clean synthetic magnitude datasets and add Rician noise to it. They also perform blind denoising, where the network is tested with a different level of noise than it was trained with. Their blind denoising test yields interesting results, since they prove that, when the network is trained with higher levels of noise and tested on lower levels of noise, the network yields better results than when training and testing with low noise.

#### 2.1.3 Unsupervised Learning

For unsupervised image denoising a novel method is presented by Xu et al. (2020), where they introduce a method that uses corrupted test images as their ground truth “clean” images. To train their network they use synthetic images consisting of small alterations to the corrupted test image. They add more noise to the test image, and they prove that if they introduce a small amount of noise to the test image as an alteration, their network is still capable of denoising the corrupt image and produce a clean output. Given their training methodology, which trains an image-specific network for each image to be denoised, their approach is not well suited for MRI denoising, given the volume of images contained in an MRI. Therefore, the denoising process would be too time-consuming.

One of the most effective models used for unsupervised denoising is presented by Soltanayev and Chun (2018) and it is based on Stein’s unbiased risk estimator, SURE. The SURE estimator, presented by Stein (1981) is an unbiased MSE estimator. The only problem with the SURE estimator is that it can only be expressed in an analytical form. When this is not available, Ramani et al. (2008) proposed a Monte-Carlo-based SURE, MC-SURE. The work presented by Soltanayev and Chun (2018) overcomes previous shortcomings and combines the Monte-Carlo approximation and makes it available for deep neural network models. Since it can be used with no need of noiseless ground truth data, deep neural networks can be trained for denoising purposes in an unsupervised manner.

The model Noise2Noise (N2N) by Lehtinen et al. (2018), saw success in denoising images by learning to predict one noisy image from another by training on independent pairs of noisy images. The result is a model that predicts the expected value of the noisy distribution for each pixel. For many real noise models, Gaussian, Poisson, etc, this expected value is clean signal.

Building upon this, Noise2Void (N2V) by Krull et al. (2018) developed a strategy which removes the need for two independent samples, and instead learns to denoise an image in a fully self-supervised way. In place of a second independent sample, N2V learns to denoise from the receptive field of a single pixel, excluding itself.

Using this strategy, Noise2Self developed a general framework for this type of denoising problem for higher dimensional spaces, and Laine et al. (2019) denoted this form of network as a “blindspot” network and provide several improvements.

Despite all the progress in unsupervised denoising in other areas, there is not that much work done in unsupervised MRI denoising. One example is by Eun et al. (2020), where they introduce a cycle generative adversarial network, CycleGAN to denoise compressed sensing MRI. Thus, we wanted to further explore this path, given the potential that unsupervised learning showed in other fields and the lack of clean ground truth data when working with MRI.

### 2.2 Background

#### 2.2.1 K-Space

In MRI terminology, k-space is the 2D or 3D Fourier transform of the MRI measured. When measuring an MRI, the complex values are sampled using a pulse sequence, such as radio-frequency and gradient pulses. At the end of the scan the data is mathematically processed to produce a final image. Therefore k-space holds raw data before reconstruction. K-space can be seen as an array of numbers representing spatial frequencies in the MRI.

**FIGURE 1:**
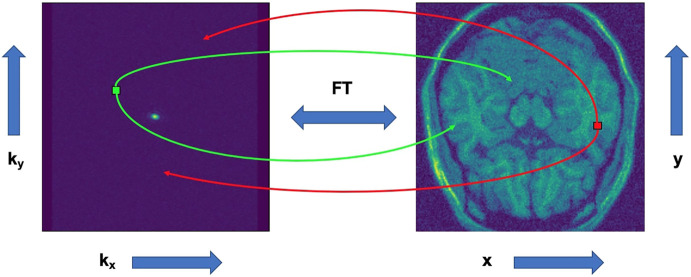
Representation of how points translate between k-space and complex image space.

To transition between k-space and the complex image space, we apply an inverse fast Fourier transform, and vice versa. Even though they are visually different, the information contained in both spaces is the exactly the same. In k-space, the axes represent spatial frequencies instead of positions. The points plotted in this space do not correspond one on one to the pixels on the image in time domain. Every point in k-space contains information about phase and spatial frequency for every pixel in the time as seen in Figure 1.

In MRI, the thermal noise that deteriorates the k-space is Gaussian. This Gaussian noise model can be defined as y=x+n, where *x* is the original MRI signal and *n* is Gaussian noise. Even after applying the inverse fast Fourier transform, the noise remains Gaussian. If we converted the complex MRI to magnitude MRI, then the noise would be Rician. This is why, we want to explore Gaussian denoising of complex-value data and avoid dealing with Rician noise in the magnitude space.

#### 2.2.2 SURE Estimator

When training a network, a gradient-based optimization algorithm is used such as the stochastic gradient descent (SGD) Bottou (1999), momentum, or the Adam optimization algorithm Kingma and Ba (2015) to optimize the loss. In our case, we use the Mean Squared Error, MSE web (2020a), to calculate the amount of noise present in the image.

$$\frac{1}{M}\sum_{j=1}^{M}{\left\|h\left(y^{\left(j\right)};\theta \right)-x^{\left(j\right)}\right\|}^{2}$$ (1)

where *M* is the number of samples in one batch of data. The main issue with Eq. 1 is that, since we are working in an unsupervised environment, we do not have access to *x*, the ground truth. Therefore, an estimator for MSE needs to be used. This is done by the SURE estimator presented in Eq. 2

$$\frac{1}{M}\sum_{j=1}^{M}\left[{\left\|y^{\left(j\right)}-h\left(y^{\left(j\right)};\theta \right)\right\|}^{2}-K\sigma^{2}+2\sigma^{2}\overset{K}{\underset{i=1}{\sum }}\frac{\partial h_{i}\left(y^{\left(j\right)};\theta \right)}{\partial y_{i}}\right]$$ (2)

noting that no noiseless ground truth data were used in Eq. 2.

The only problem with the SURE estimator is that the last divergence is intractable. However it can be approximated using the Monte-Carlo SURE estimator by Ramani et al. (2008). Therefore the final risk estimator which will be used as a loss function is

$$\frac{1}{M}\overset{M}{\underset{j=1}{\sum }}\left\{y^{\left(j\right)}-h{\left(y^{\left(j\right)};\theta \right)}^{2}-K\sigma^{2}+\frac{2\sigma^{2}}{\epsilon }{\left({\tilde{n}}^{\left(j\right)}\right)}^{\text{t}}\left[h\left(y^{\left(j\right)}+\epsilon {\tilde{n}}^{\left(j\right)};\theta \right)-h\left(y^{\left(j\right)};\theta \right)\right]\right\}$$ (3)

where ε is a small fixed positive number and n∼(j) is a single realization from the standard normal distribution for each training data *j*.

#### 2.2.3 Blindspot Network

Laine et al. (2019) provide an improved blindspot architecture and denoising procedure. The blindspot network architecture combines multiple branches, where each branch restricts its receptive field to a half-plane which does not contain the center pixel. Then four branches are combined using 1×1 convolutions. This form allows for the receptive field to be efficiently extended arbitrarily in every direction, while still excluding the center pixel.

In N2V, the center pixel information is not exploited to prevent the model from simply learning to output this value. However, using Bayesian reason to the denoising task, we have for a particular noisy pixel *y* and corresponding clean signal *x*

$$p\left(x\;|\;y,{\text{Ω}}_{y}\right)\propto p\left(y\;|\;x\right)p\left(x\;|\;{\text{Ω}}_{y}\right)$$ (4)

where ${\text{Ω}}_{y}$ is the context given by the receptive field of the pixel *y*. Thus, using a blindspot architecture to model a Gaussian prior $p\left(x\;|\;{\text{Ω}}_{y}\right)$, the posterior mean $E_{x}\left[p\left(x\;|\;y,\text{Ω}\right)\right]$ has a closed form solution for many noise models. This allows for the use of the previously unexploited center pixel data at test time. In the case of MRI, with a Gaussian noise model, the posterior mean can be computed analytically.

#### 2.2.4 Datasets

##### 2.2.4.1 Knee MRI

The Center for Advanced Imaging Innovation and Research (CAI^2^R), in the Department of Radiology at New York University, NYU, School of Medicine and NYU Langone Health, released two MRI datasets, Zbontar et al. (2018), Zbontar et al. (2020), to work on rapid image acquisition and advanced image reconstruction. The deidentified datasets consist of scans of knees and brains, which contain raw k-space data. For this experiment, we decided to use single coil data only, as it is the most widely used modality and due to its data size compared to multi coil, which is smaller.

The knee single coil dataset contains 973 training subjects and 199 validation subjects. According to their website, the fully sampled knee MRIs were obtained on 3 and 1.5 Tesla magnets. The raw dataset includes coronal proton density-weighted images with and without fat suppression. As such, NYU fastMRI investigators provided data but did not participate in analysis or writing of this report. A listing of NYU fastMRI investigators, subject to updates, can be found at web (2020b).

Note that all knee MRI contain noise that varies from subject to subject.

##### 2.2.4.2 Brainweb

In most of today’s image analysis methods, a ground truth is expected, even if just for validation. In the case of MRI, noise is implicit to the *in vivo* acquisition process, and so no true noise free MR dataset exists. The Brainweb dataset provides an easy solution for this by creating a Simulated Brain Database (SBD) Cocosco et al. (1997); web (1998); Kwan et al. (1999); Kwan et al. (1996); Collins et al. (1998), where an MRI simulator is used to created realistic MRI data volumes. In addition to providing a predefined magnitude image dataset, the Brainweb simulator is exposed to allow for custom simulations.

Using the custom simulator, we acquired raw frequency spatial data for varied simulator parameters. This includes data generated for all combinations of no, mild, moderate, and severe multiple sclerosis (MS) lesions anatomic models with the six available parameter templates. These six are generated by combining the AI and ICBM protocols with either T1, T2, or Proton Density (PD) weighting. For our purposes, we will only be using T1 and T2. All together this allowed for the generation of 16 brain MR volumes simulated from a realistic parameter set. 12 subjects were used for training and four subjects were used for testing. Additionally, the custom simulator allows for adding a noise level; however, as we are treating this data as ground truth, we did not use this feature. For all Brainweb experiments, we performed cross-validation to ensure the validity of the results.

Since our blindspot network expects square input, each individual slice of the MR volumes were zero padded in k-space to have matching dimensions.

### 2.3 Training

All models were trained and tested using a single NVIDIA GeForce GTX Titan X, with 12 GBytes of memory.

#### 2.3.1 SURE Model

The gradient of Eq. 3 can be automatically calculated when training a deep learning framework. Therefore, we use Eq. 3 as a cost function for a basic U-Net architecture, Ronneberger et al. (2015), with five convolutional layers on both sides.

To train the SURE estimator in 2D, we use a U-Net of depth 5, convolution kernel size of 3 and 48 initial feature maps. After each convolutional layer, a LeakyReLU is applied, except for the last convolutional layer, where no activation function is used. We train the network in batches of 10 for 300 epochs, using the Adam optimizer with an initial learning rate of $3\times 10^{-4}$. The data, both training and testing, is center cropped to 320 × 320 for knee MRI and 192 × 192 for brain MRI, using all available slices for both.

#### 2.3.2 Blindspot Model

Due to large regions of no-signal in MRI and a shared standard deviation across all pixels, many techniques exist to estimate the standard deviation of the noise *σ*, Sardy et al. (2001). Thus, we use a blindspot architecture with knowledge of *σ*, and our prior becomes $p\left(x\;|\;{\text{Ω}}_{y},\sigma \right)$. This modifies Eq. 4 in training to

$$p\left(x\;|\;y,{\text{Ω}}_{y}\right)\propto p\left(y\;|\;x\right)p\left(x\;|\;{\text{Ω}}_{y},\sigma \right)$$

We train a 5-layer deep blindspot network in batches of 5 for 300 epochs. The convolution kernel has size of 3 and there are 48 initial feature maps. No activation function is used. We use Adam optimizer with an initial learning rate of $3\times 10^{-4}$. The learning rate is reduced if the validation loss has not decreased after ten epochs. The data, both training and testing, is center cropped to 320 × 320 for knee MRI and 192 × 192 for brain MRI, using all available slices for both. For a more detailed network architecture description, please refer to Laine et al. (2019). We used the same blindspot network and U-Net architecture as described in Laine et al. (2019).

## 3 Results

For both datasets, different levels of noise were added to the original images in order to do a quantitative comparison to NLM. Since both models rely on Gaussian noise, we will only be adding Gaussian noise to the images.

**FIGURE 2:**
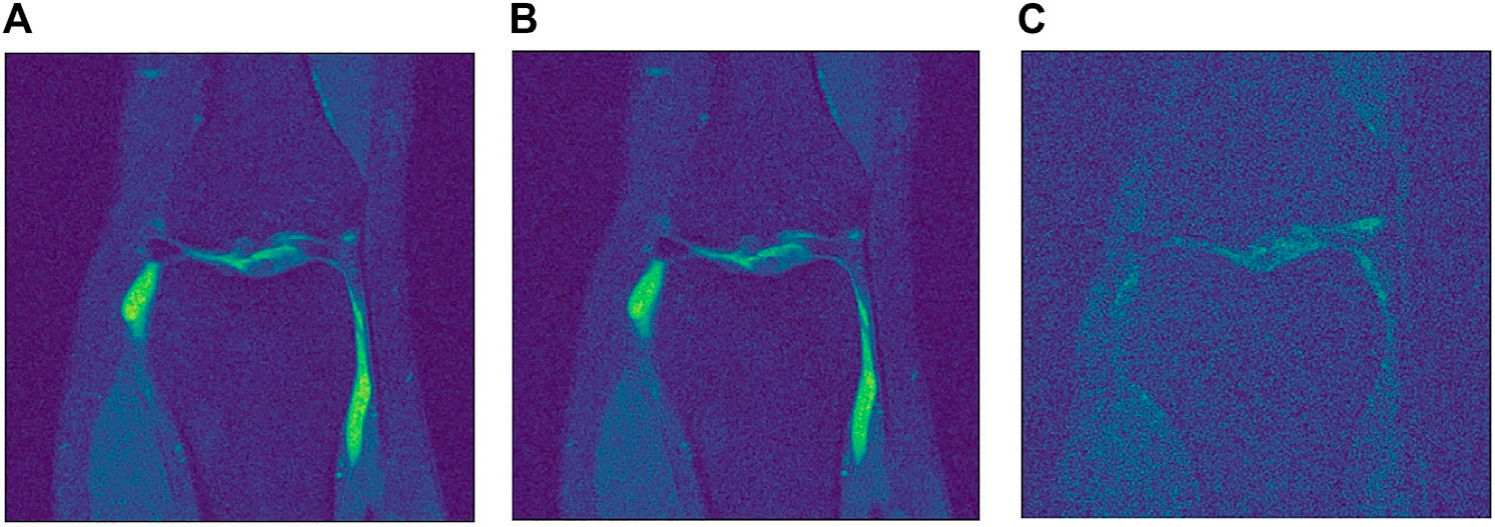
Different levels of noise. **(A)** Low level $\sigma =8.2\times 10^{-6}$. **(B)** Medium level $\sigma =1\times 10^{-5}$. **(C)** High level $\sigma =2\times 10^{-5}$.

For the knee single coil dataset, we started by adding noise with $\sigma =1\times 10^{-5}$. Then, we followed with twice the amount of noise with $\sigma =2\times 10^{-5}$ to test both algorithms with an elevated amount of noise. Finally, the average background noise, $\sigma =8.2\times 10^{-6}$, was calculated for all images and was used for the last test. The three levels of noise can be seen in Figure 2. Since the data is comprised of small values, a scale factor is needed. This factor is calculated using the maximum value found in the dataset as a reference. For both networks, a scale factor of 500 was used.

**FIGURE 3:**
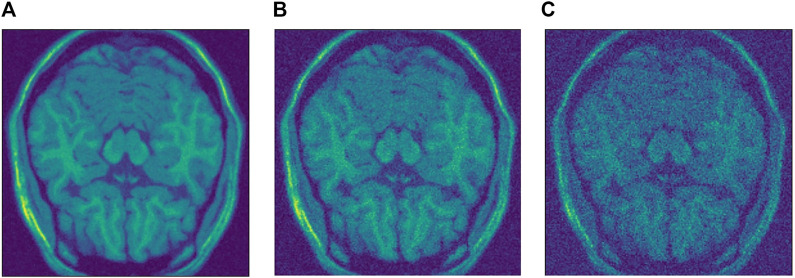
Different levels of noise. **(A)** Low level σ=50. **(B)** Medium level σ=100. **(C)** High level σ=200.

For the Brainweb dataset, we added three different levels of noise. To understand how the networks behave with different levels of noise, we used low level noise with σ=50, middle noise with σ=100 and high level noise with σ=200. In this case, since the data has much bigger values, a higher sigma is used. The three levels of noise can be seen in Figure 3. Note how the data has to be scaled too, specially for the SURE network, which is highly sensitive to the input scale. For the Brainweb dataset, we scaled all input by a factor of 1/25,000. While the blindspot network presented good results even without the scaling factor, it performed slightly better with scaling.

In order to evaluate the proposed algorithm, three quantitative measures were used for the first three tests. Through all tests, a qualitative measure will be used, based on our perception of the images.

The three quantitative measures used are peak signal-to-noise-ratio, PSNR, mean-squared error, MSE web (2020a) and Structural Similarity Index Measure, SSIM Wang et al. (2004). Both MSE and PSNR are used to compare image compression quality, while SSIM is used for measuring the similarity between two images.

MSE represents the cumulative squared error between the compressed and the original image. The lower the value of MSE, the lower the error. MSE can be defined as

$$MSE=\frac{\sum_{M,N}{\left[I_{1}\left(m,n\right)-I_{2}\left(m,n\right)\right]}^{2}}{M*N}$$ (5)

where *M* and *N* are the number of rows and columns in the input image.

PSNR computes the peak signal-to-noise ratio between two images. This ratio is used as a quality measurement between the original and a compressed or reconstructed image. The higher the PSNR, the better the quality of the image. PSNR can be defined as

$$PSNR=10{\text{log}}_{10}\left(\frac{MAX^{2}}{MSE}\right)$$ (6)

where MAX is the maximum achievable value in the input image data type.

SSIM is a method for measuring the similarity between two images. The SSIM index can be viewed as a quality measure of one of the images being compared, taking into account that the other image is regarded as of the ground truth.

The main difference between SSIM and PSNR or MSE is that SSIM quantifies the change in structural information, while PSNR or MSE approach estimate absolute errors. Structural information, such as luminance and contrast, is based on the fact that pixels have inter-dependencies, especially when they are spatially close.

The overall index is a multiplicative combination of the three terms and can be described the following way:

$$\text{SSIM}\left(x,y\right)={\left[l\left(x,y\right)\right]}^{\alpha }\cdot {\left[c\left(x,y\right)\right]}^{\beta }\cdot {\left[s\left(x,y\right)\right]}^{\gamma }$$ (7)

where

$$\begin{array}{c} l\left(x,y\right)=\frac{2\mu_{x}\mu_{y}+C_{1}}{\mu_{x}^{2}+\mu_{y}^{2}+C_{1}},\\ c\left(x,y\right)=\frac{2\sigma_{x}\sigma_{y}+C_{2}}{\sigma_{x}^{2}+\sigma_{y}^{2}+C_{2}},\\ s\left(x,y\right)=\frac{\sigma_{xy}+C_{3}}{\sigma_{x}\sigma_{y}+C_{3}} \end{array}$$ (8)

where $\mu_{x}$, $\mu_{y}$, $\sigma_{x}$, $\sigma_{y}$ and $\sigma_{xy}$ are the local means, standard deviations, and cross-covariance for images *x*, *y*. If *α* = *β* = *γ* = 1, and $C_{3}=C_{2}/2$ the index simplifies to:

$$\text{SSIM}\left(x,y\right)=\frac{\left(2\mu_{x}\mu_{y}+C_{1}\right)\left(2\sigma_{xy}+C_{2}\right)}{\left(\mu_{x}^{2}+\mu_{y}^{2}+C_{1}\right)\left(\sigma_{x}^{2}+\sigma_{y}^{2}+C_{2}\right)}$$ (9)

For all the results that are presented here, an optimal h parameter for the NLM algorithm was previously found and set to *h* = 0.71. The patch size was set to 5 × 5 with a patch distance of 6.

The same tests were done for both the SURE network and the blindspot network, Table 1, 2 respectively. For each evaluation metric, the best scoring algorithm is highlighted in bold.

**TABLE 1:** Test results knee single-coil dataset.

*σ*	Noisy MSE	SURE MSE	Blindspot MSE	NLM MSE
$8.2\times 10^{-6}$	$6.5954\times 10^{-11}$	$3.6943\times 10^{-11}$	$3.9075\times 10^{-11}$	$3.9826\times 10^{-11}$
$1\times 10^{-5}$	$9.8777\times 10^{-11}$	$4.7123\times 10^{-11}$	$4.8734\times 10^{-11}$	$4.9732\times 10^{-11}$
$2\times 10^{-5}$	$4.2101\times 10^{-10}$	$9.0616\times 10^{-11}$	$8.7264\times 10^{-11}$	$9.0004\times 10^{-11}$

*σ*	Noisy PSNR	SURE PSNR	Blindspot PSNR	NLM PSNR
$8.2\times 10^{-6}$	28.266	30.866	30.626	30.555
$1\times 10^{-5}$	26.512	29.846	29.692	29.610
$2\times 10^{-5}$	20.226	27.196	27.329	27.235

*σ*	Noisy SSIM	SURE SSIM	Blindspot SSIM	NLM SSIM
$8.2\times 10^{-6}$	0.7238	0.7795	0.7708	0.7661
$1\times 10^{-5}$	0.6487	0.7284	0.7215	0.7119
$2\times 10^{-5}$	0.3628	0.5579	0.5605	0.5653

**TABLE 2:** Test results for the Brainweb dataset.

*σ*	Noisy MSE	SURE MSE	Blindspot MSE	NLM MSE
50	2,981.044	1,281.977	1,259.961	1,322.726
100	12,332.774	3,508.540	2,758.001	4,059.259
200	50,639.730	9,150.021	7,245.904	11,578.606

*σ*	Noisy PSNR	SURE PSNR	Blindspot PSNR	NLM PSNR
50	33.524	38.015	38.012	37.781
100	27.361	34.036	35.240	33.166
200	21.227	30.301	31.429	28.753

*σ*	Noisy SSIM	SURE SSIM	Blindspot SSIM	NLM SSIM
50	0.7663	0.8971	0.8977	0.8790
100	0.6314	0.8466	0.9066	0.8014
200	0.4710	0.7829	0.8409	0.6996

## 4 Discussion

As seen in Table 1 for the knee data, the SURE network presents better results than NLM and blindspot for both $\sigma =1\times 10^{-5}$ and $\sigma =8.2\times 10^{-6}$. In both those cases, MSE is smaller and both PSNR and SSIM are larger than NLM and blindspot. Note how in the case of $\sigma =2\times 10^{-5}$, NLM does better than the SURE network, but worse than blindspot, except for SSIM. Given that this is an extreme case, where the amount of noise is unrealistically elevated, it would be uncommon to find data in those circumstances.

We can also see that the blindspot network presents better results than NLM for all levels of noise, except for SSIM for $\sigma =2\times 10^{-5}$. Compared to SURE, it presents worse results for $\sigma =1\times 10^{-5}$ and $\sigma =8.2\times 10^{-6}$. Note however, how in the case of $\sigma =2\times 10^{-5}$, blindspot outperforms both SURE and NLM except for NLM SSIM. This presents a divergence in the results previously seen in the complex image space, where for the case of high level noise, NLM was overall better than blindspot and SURE.

For the Brainweb dataset, both networks present better results in all scoring metrics than NLM. The best overall performing network is the blindspot network, edging out the SURE network, except in one case, PSNR for σ=50, where SURE is slightly better than blindspot. Again, we believe that in this case both networks do better than NLM even in the presence of high amounts of noise because there is no background noise at all in the original images. Therefore, the networks only need to remove just the added noise.

**FIGURE 4:**
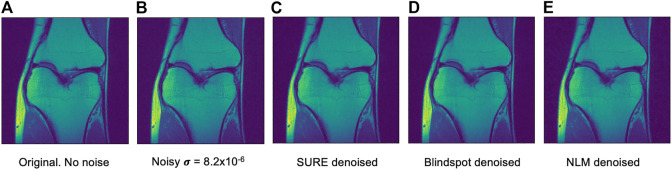
Example of denoised knee MRI for $\sigma =8.2\times 10^{-6}$. The example image is the middle slice from one of the subjects. In this case, this is the PSNR for every method for this particular subject. **(A)** Original image, no noise added—**(B)** Noisy image—**(C)** SURE PSNR = 37.092—**(D)** Blindspot PSNR = 37.317—**(E)** NLM PSNR = 36.350.

**FIGURE 5:**
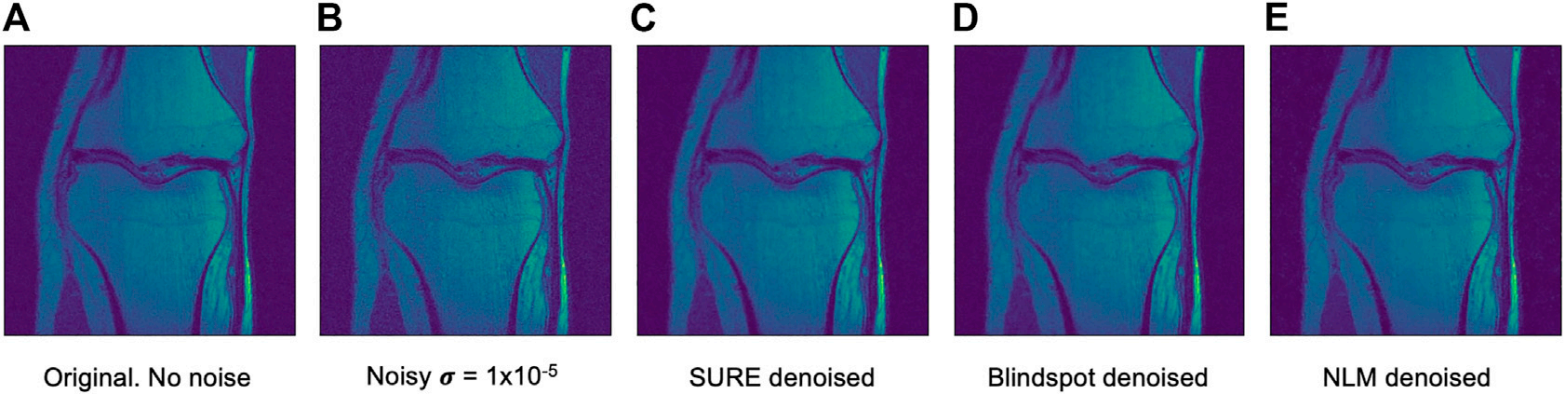
Example of denoised knee MRI for $\sigma =1\times 10^{-5}$. The example image is the middle slice from one of the subjects. In this case, this is the PSNR for every method for this particular subject. **(A)** Original image, no noise added—**(B)** Noisy image—**(C)** SURE PSNR = 30.800—**(D)** Blindspot PSNR = 30.953—**(E)** NLM PSNR = 30.189.

**FIGURE 6:**
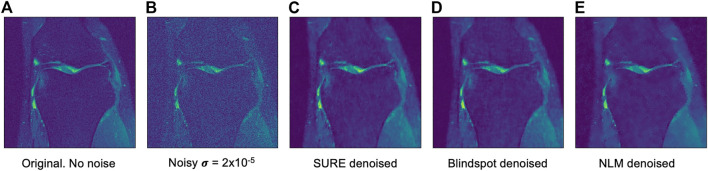
Example of denoised knee MRI for $\sigma =2\times 10^{-5}$. The example image is the middle slice from one of the subjects. In this case, this is the PSNR for every method for this particular subject. **(A)** Original image, no noise added—**(B)** Noisy image—**(C)** SURE PSNR = 23.823—**(D)** Blindspot PSNR = 23.931—**(E)** NLM PSNR = 24.086.

Another comparison can be done using qualitative measures, based on observing the images and comparing all outputs. Using Figures 4–6 as references, at a first glance, NLM does a better job at taking noise out, but does it while having a negative effect on the edges and the tissue pixels. NLM does an excellent job when removing noise from the background, but does not do as well on the tissue pixels. This can be a problem, since we want to maintain the tissue structure as much as possible. The SURE network does a better job at preserving the tissue while doing a good job when denoising. In some cases, NLM introduces artifacts that interfere with the tissue pixels. In terms of edge preservation, again NLM presents an undesired effect, which makes the edges look worse than the original image.

**FIGURE 7:**
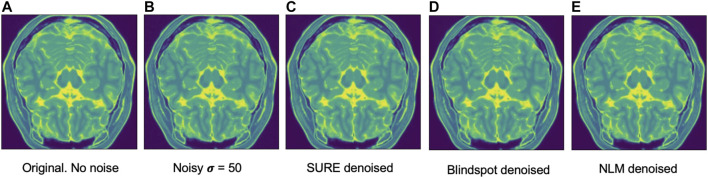
Example of denoised brain MRI for σ=50. The example image is the middle slice from one of the subjects. In this case, this is the PSNR for every method for this particular subject. **(A)** Original image, no noise added—**(B)** Noisy image—**(C)** SURE PSNR = 43.883—**(D)** Blindspot PSNR = 44.731—**(E)** NLM PSNR = 43.000.

**FIGURE 8:**
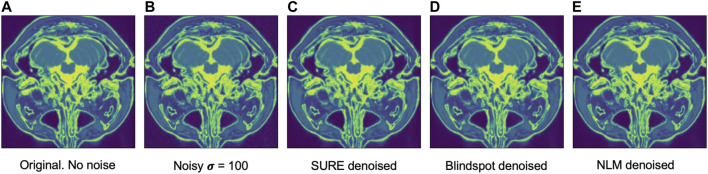
Example of denoised brain MRI for σ=100. The example image is the middle slice from one of the subjects. In this case, this is the PSNR for every method for this particular subject. **(A)** Original image, no noise added—**(B)** Noisy image—**(C)** SURE PSNR = 38.130—**(D)** Blindspot PSNR = 39.072—**(E)** NLM PSNR = 37.108.

**FIGURE 9:**
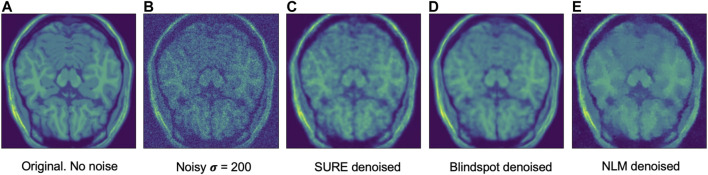
Example of denoised brain MRI for σ=200. The example image is the middle slice from one of the subjects. In this case, this is the PSNR for every method for this particular subject. **(A)** Original image, no noise added—**(B)** Noisy image—**(C)** SURE PSNR = 29.610—**(D)** Blindspot PSNR = 30.904—**(E)** NLM PSNR = 26.616.

**FIGURE 10:**
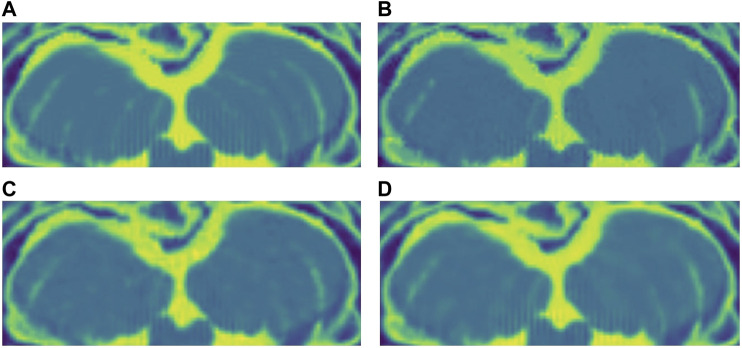
**(A)** Original close-up. No noise added. **(B)** NLM denoised close-up. **(C)** SURE network denoised close-up. **(D)** Blindspot denoised close-up. Observe how all three algorithms do a good job at denoising, but NLM introduces undesired artifacts.

**FIGURE 11:**
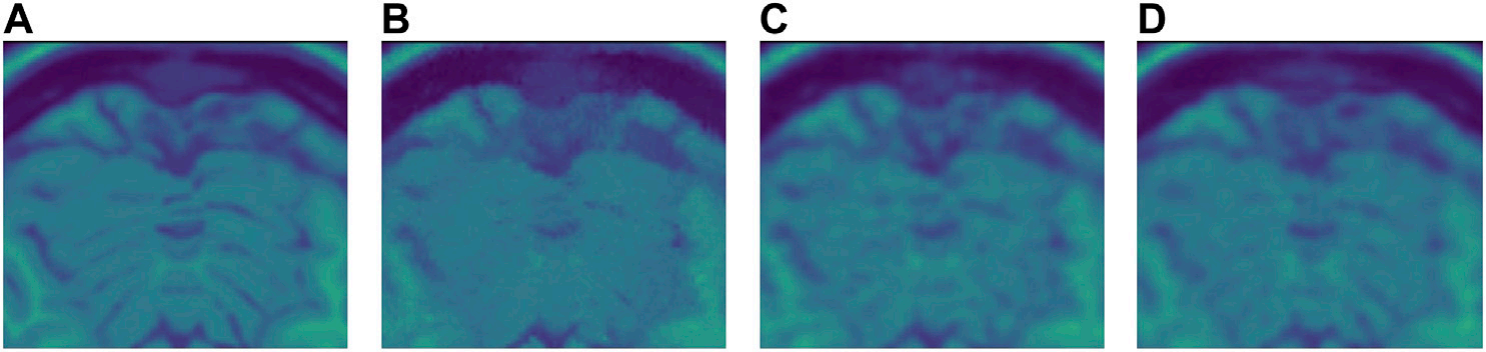
**(A)** Original close-up. No noise added. **(B)** NLM denoised close-up. **(C)** SURE network denoised close-up. **(D)** Blindspot denoised close-up. Observe how NLM completely removes some of the tissue while both SURE and blindspot, do not remove as much noise, but do a better job at maintaining the tissue’s structure without inserting any artifacts.

For the Brainweb dataset, both networks present better results in all scoring metrics than NLM. The best overall performing network is the blindspot network, edging out the SURE network. We believe that in this case both networks do better than NLM even in the presence of high amounts of noise because there is no background noise at all in the original images. Therefore, the networks only need to remove just the added noise. We can see this in Figures 7, 8, 9. NLM still presents an undesired effect on the images which can be costly. If we take a closer look, we can see some of the tissue details that the NLM is removing completely and some of the artifacts that it presents. We can clearly see this in Figures 10, 11.

After seeing how both networks outperform NLM in most categories, the next step was to work with the original images from the knee dataset, without adding any extra noise. When doing this test, no quantitative measure can be used, since there is no image to compare to. Therefore, only qualitative measures will be used.

**FIGURE 12:**
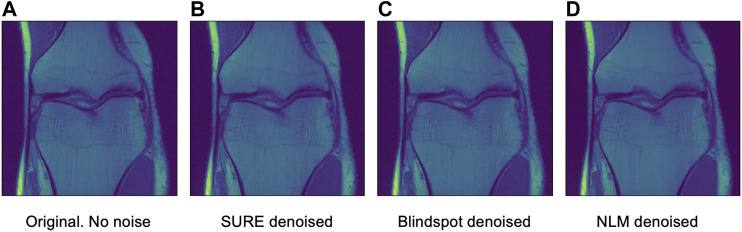
Example 1 of denoised brain MRI without adding any noise. The example image is the middle slice from one of the subjects. **(A)** Original image, no noise—**(B)** SURE denoised image—**(C)** Blindspot denoised image—**(D)** NLM denoised image.

**FIGURE 13:**
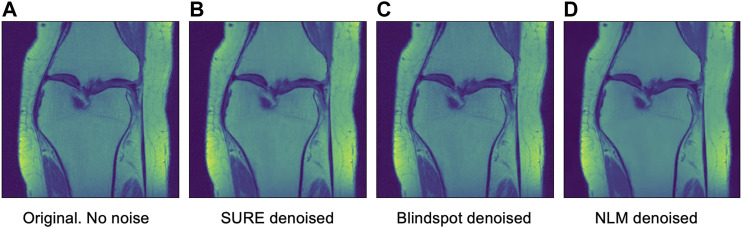
Example 2 of denoised brain MRI without adding any noise. The example image is the middle slice from one of the subjects. **(A)** Original image, no noise—**(B)** SURE denoised image—**(C)** Blindspot denoised image—**(D)** NLM denoised image.

As seen in Figures 12, 13, both networks have mixed results. Both networks still do a better job at preserving the edges and tissue, but sometimes struggle to remove noise from parts of the image without any tissue. This is happening due to a few circumstances. First of all, when training the data, there is no ground truth to compare it to. This can lead to over-training and over-fitting. Second, the inherent noise that the images have, might not be Gaussian noise. This is also supported by the previous results that were obtained for both datasets. Both the SURE and blindspot network were outperformed only in the presence of high levels of noise for the knee dataset. In the same conditions of high level of noise for the Brainweb dataset, both networks outperformed NLM. Therefore, the background noise from the knee dataset has a negative effect on the networks, which might indicate that it is not truly Gaussian. The discrepancy in the type of noise might also be causing the calculated *σ* to be irrelevant and misleading, since *σ* is used for both networks. Despite all of this, the networks are competitive with NLM in most cases.

## 5 Conclusion

We evaluated two unsupervised approaches to denoise Magnetic Resonance Image, MRI, one approach based on a Stein’s Unbiased Risk Estimator and another one based on a Blindspot network. Using the complex image space, innate to MRI, we tested a real dataset containing knee MRI, and a synthetic dataset consisting of brain MRI. Both networks were compared against Non-Local Means using quantitative and qualitative measures. Both networks outperformed NLM for all scoring metrics except when in the presence of exceptionally high levels of noise. One interesting direction that we would like to explore is 3D denoising using both networks. This is especially compelling for the blindspot network, since we will have to explore a 3D receptive field.

## Data Availability

The data analyzed in this study is subject to the following licenses/restrictions: Need to ask for personalized download code from dataset owners. Requests to access these datasets should be directed to fastmri@med.nyu.edu.
